# Discussion on Inversion of the Uterus, in the New York State Medical Society

**Published:** 1861

**Authors:** 


EXTRACTS FROM THE PROCEEDINGS OF THE NEW YORK STATE MEDICAL SOCIETY,

Published in the *American Medical Times*,

### DISCUSSION ON INVERSION OF THE UTERUS.

We quote the following discussion, partly for its intrinsic value, and partly for the purpose of correcting some errors of fact alluded to therein. For instance: the reader will see that Dr. C. A. Lee, in his remarks alludes to the case of Fisher vs. Stone, which was tried in this city, and assumes that the patient, Mrs. S., had "*oft-repeated hemorrhages during all that interval*," of thirty or forty days intervening between the confinement and the discovery of the inversion.

And on this assumption, he distinctly charges that Dr. Fisher "*was guilty of neglect to his patient in not making an examination sooner to find out the cause of the hemorrhage*."

Dr. Lee not being present at the trial, and the testimony in relation to the facts of the case of Dr. Fisher having never been published, we are at a loss to know on what authority he states that "oft-repeated hemorrhages" occurred during all the thirty or forty days. We cannot help thinking that every rule of propriety, forbids the attempt to censure a physician publicly and in print, especially under circumstances when he has no possible chance to defend himself, except on reliable and well established evidence. But so far from the allegation that "*oft-repeated hemorrhages*" occurred during *all* the thirty or forty days spoken of, being an established fact; it was an allegation positively denied by unimpeached and unimpeachable testimony on the trial. Hence, we feel called upon to say, that the remarks of Dr. Lee, in the New York State Medical Society on this point, are not based on sufficient evidence, and are therefore unjust to an intelligent and honorable member of the profession.—[Ed. Examiner.]

Dr. A. Van Dyck read a paper entitled "Inversion of the Uterus," which elicited the following discussion:

Dr. Quackenbush stated that a very interesting case of the sort came up for trial in Chicago not long since ; the physician, Dr. Fisher, being charged with malpractice. It seemed that not until three weeks after the confinement was anything of the kind noticed, and the parts were not replaced until three or four months had elapsed. The jury came to the conclusion that the organ became gradually inverted, and after remaining in the vagina all that time, finally passed out. The case was decided against the physician, notwithstanding, it was proved that the vagina was at that time so far unoccupied as to allow the passage of a large syringe into it for the purpose of injection. The nurse also affirmed that the uterus was firmly contracted after delivery.

Dr. Thorne mentioned a case which occurred to him some years ago. He was consulted by a woman lately from England, who presented between her thighs a tumor covered with a cuticle, resembling the skin in appearance. She stated that she had been afflicted by its presence for a number of years, but desired treatment more particularly at that time in consequence of the presence of a troublesome ulceration upon its surface. After very careful examination the tumor was decided to be an inverted uterus. Dr. Blatchford also saw the case, and upon consultation it was deemed best to attempt a reduction, which was effected without much difficulty. In answer to Dr. Quackenbush he stated that there was some dimpling of the organ.

Dr. Quackenbush referred to a case of inversion seen by Dr. White, where, after an existence of thirteen years, reduction was effected. He thought that in all cases of inversion restitution took place at the os, and continued from below upwards, instead of by simple dimpling of the fundus.

Dr. Van Dyck thought that some distinction should be made between recent and old standing cases, inasmuch as in the latter instances it would be almost impossible to produce that dimpling. In his case he tried to shove up the organ with his whole hand, but could not succeed. He then lengthened out the finger, soon the dimpling came on, and the organ was replaced. In some of these he thought that dimpling was a matter of necessity.

Dr. Blatchford stated, that soon after seeing the case with Dr. Van Dyck, he dined with Dr. Wing, who stated that he had seen just such a case, and reduced it in the same way.

Dr. Quackenbush in answer to an inquiry from Dr. McNulty, stated that he did not believe inversion could take place unless the body of the uterus has been previously filled, either by a fœtus or a tumor. The uterus in its normal state had too small an amount of muscular fibre to render any such effect possible, and, besides, the cavity of the organ was so small that there was very little or nothing to be inverted.

Dr. McNulty asked Dr. Q. if he believed any predisposition ever existed in any given case, and whether inversion might not take place, independent of any mechanical cause?

Dr. Quackenbush.—I do not think that that question has been much studied; the impressson is, whenever we have these cases of inversion, and when we can't apply a better reason, that there is a predisposition. Authors state this to be a fact, but what they mean by it, it is hard to tell. My impression is that inversion takes place sometimes without any mechanical cause. It has been generally supposed that injudicious treatment could only produce it, yet we find such a state of things occurring occasionally in the practice of the most scientific obstetricians, when there has been no drawing upon the cord whatever, and sometimes in fact, before there has been any endeavor on the part of the obstetrician to remove the placenta at all. In some cases you draw very lightly upon the cord and the womb becomes inverted, while in others you may pull with a great deal of force without producing the accident. In order to explain this, it is necessary to assume that some pathological condition, which we call a predisposition, exists in one instance and not in another.

Dr. Van Dyck asked Dr. Q. if there was any such thing as a gradual inversion, remarking that such was the doctrine taught in the books.

Dr. Quackenbush said that a great deal was taught in the books which was absolutely wrong, and in the matter of inversion, this was especially the case. He maintained that in cases of inversion, the succession of events was generally so rapid, that it was impossible to form an idea exactly how it took place. He remarked that bookmakers were too apt to follow implicitly the assertions of those who had preceded them in the task, without stopping to inquire the foundation for such assertions.

Dr. Brinsmade asked Dr. Lee to favor the Society with a few remarks upon the subject under consideration, but there being little time left in the morning session, it was agreed to assign to Dr. Lee the first part of the afternoon session.

The Committee on Credentials made a partial report, after which, on motion of Dr. McNulty, the meeting adjourned until 3¼ P. M.

AFTERNOON SESSION.

The meeting was called to order by the President, Dr. Jones, when the minutes of the previous meeting were read by the Secretary, Dr. S. D. Willard, and approved.

Dr. C. A. Lee said:—I do not claim to know much more on this subject of inversion of the womb than the profession generally, and the reason why I have been called to make some remarks upon this occasion is, owing to the fact that a paper was published by me in the October number of the *American Journal of the Medical Sciences.* I was led to study the subject, as I was called upon to make a deposition in the Chicago case in which there were fifty questions put to me. I found quite a large number of cases scattered through the books; I collected about two hundred, and made an analysis of one hundred and forty-eight. I do not claim to know very much of the subject from practical experience. I presume there are physicians in this room who have been in midwifery practice all their lives, and yet have never met with a case. It is well known in the London and Dublin Lying-in Hospitals that out of 40,000 cases of delivery, not a single case of inversion has occurred. I infer from that fact, that this accident does not often occur in the practice of physicians well acquainted with the science of midwifery, and that it is due to carelessness and inattention on the part of the attendant: I will not say want of skill, because a sudden issue may take place and the womb become inverted before we suspect any danger. We know that this happened to Professor Dewees, and with that ingenuousness which was a characteristic of the man, he relates how, in attending a case, before he suspected that anything was going to happen, the placenta came down adherent to the fundus, that the womb was inverted, and then he goes on to describe how he replaced it. You will find that Dr. Ramsbotham also describes a case which occurred in his practice which he thought was owing to negligence on his part. There is a belief which is very prevalent in the profession, that when the womb is inverted and placenta adherent, it must make its appearance externally. This I believe to be a mistake; certainly after the placenta is separated there is no room in the vagina for the womb to remain without being visible externally. In a letter from Professor White, of Buffalo, which I have received within a few days, he describes a case where he found the uterus in this condition.

In regard to the manner in which this takes place, I consider it rather a matter of theory. No man has ever observed it, for if he had been watching for it, it would not have occurred. Some say it is due to irregular contraction; some say, with Dr. Quackenbush, that evolution commences first at the neck of the womb; others say that the fundus is dimpled and gradually projects downwards, until at length the womb takes the alarm and begins to expel the fundus exactly as it would a foreign body. That I believe to be the way in which it generally happens. I don't believe in this evolution at the neck. To say that such a tissue can take on contraction with such a small amount of muscular fibres, so as to invert it, is absurd; however, it is a matter of pure theory. Not exactly all theory either, because you find in two cases which are quoted by Dr. Dewees in his midwifery, where the womb had been inverted, that the physicians replaced it, and suddenly as he withdrew the hand the fundus followed it down and came out externally. He then pushed the fundus up and tried to retain it there, but every time he withdrew his hand the womb came down. He thus observed that the process commenced at the fundus and not at the neck. Here, then, we have two or three actual observations to base against the theory advanced. I doubt very much whether it can be accomplished by irregular contraction. I believe that in order to invert the womb, the placenta should be adherent, the uterus relaxed, and that there should be a strong abdominal nisus, when the placenta will drag down the fundus.

Now, in regard to this case at Chicago. It is a remarkable fact that we have no instance of a medico-legal record of any such case. (The Dr. then read an abstract of the case as published.)

Now, gentlemen, in regard to the defence in this case, it was thought that the inversion took place gradually; that there was a slight dimpling of the fundus which slowly increased, and in the course of thirty or forty days the uterus turned itself inside out. The inversion occurred rather suddenly. The physicians then made the examination for the first time after her delivery, notwithstanding, there had been oft repeated hemorrhages during all that interval. Dr. Potter was afterwards consulted, who did not give a very favorable opinion of the case. Dr. Fisher heard of it, and immediately commenced a suit for slander. Mr. Stone, the husband of the lady, employed two lawyers, who proposed that Dr. Fisher should leave the case in the hands of three medical gentlemen. This proposition was not acceded to; the suit was commenced; the damages were laid at $20,000, and the case was decided against Dr. Fisher. I gave my deposition to the effect that it was not a case of gradual inversion, but that the occurrence took place at most the first or second day after delivery, and that the Doctor was guilty of neglect to his patient in not making an examination sooner to find out the cause of the hemorrhage.

In conclusion, I will allude to two or three results which I have deduced from the statistics. In regard to inversion of the womb I have shown that in sixty-two cases where the cause was assigned, thirty-nine are stated to have occurred from pulling on the cord, and I believe with Dr. Lee, of London, that this is the most frequent cause of the difficulty, especially in case the placenta is adherent. In twenty cases out of one hundred and forty-eight, labor was natural and slow, but in the majority there were symptoms of uterine exhaustion. That is another of the circumstances which I believe favors inversion. The womb is like a wet rag, and falls downwards from the mere weight of the fundus, and not by contraction of the neck. Dr. Lee, of London, says that inversion is frequently, if not invariably owing to pulling of the cord before the uterus has had time to contract. Now you hear a great deal of spontaneous evolution, when it has occurred without interference on the part of the attendant, but this I believe is very seldom the case. I do not say that it is an impossibility; but when such a case turns out, you may depend upon one thing, that is, that it commences at the time of delivery. Nor is the inversion gradual; it probably happens the first day after delivery. This was in all probability the fact in the case reported this morning; the inverted womb remained in the vagina, which, having just previously passed a fœtus, was ample enough to contain the displaced organ without having it appear externally. This point caused a great deal of discussion at Chicago, and it was contended by many, that because the uterus did not appear externally, therefore the inversion did not take place at the time of delivery. I will relate a case which occurred to me recently. I was called to a lady who had been out of health for twenty-five years, and during that time she consulted a dozen different physicians. Since the commencement of her illness she had been suffering from repeated hemorrhages, which left her in a very anæmic condition. At the time of her delivery, twenty five years ago, the midwife who attended her, used a good deal of force in taking away the afterbirth. I examined her at once, suspecting that something was the matter with her womb, either polypus or inversion. I passed my hand up and felt a body hanging down like a fibrous tumor attached to the fundus, but I did not make up my mind as to its precise character. On examining again, however, I ascertained that the womb was inverted. I wrote to Dr. White a history of the case, and asked his opinion, she being at the "change of life," whether he thought the hemorrhage would cease after that period. The Doctor thought that the flow would not be checked by delaying, and advised me to attempt a reduction. I, however, did not follow his advice. The "change of life"

brought with it a stoppage of the hemorrhage, and for the last two years the woman has enjoyed very good health. The anæmia was removed by diet. A very important inference can be drawn from this case in reference to the influence that may be exerted by the cessation of the menses. None of the physicians who had previously seen her, had made an examination to ascertain the source of the hemorrhage.

In the one hundred and forty-eight cases, the uterus was reposited in fifty-one, and three are recorded by Meigs as cases of spontaneous involution. I think Meigs made a mistake in the diagnosis; such mistakes have been made by as great men as he, by Baudelocque, Sir Charles Bell, Rigby and others. In most of the cases the placenta was separated before it was replaced. I will not detain you to speak as to whether the womb should be reduced with the placenta adherent or not; the course to be pursued must depend on circumstances. There is but one fact which I have proved, and which will, no doubt, be interesting, that is, that it is not a dangerous operation to remove the inverted uterus by ligature. I, myself, was certainly astonished to find how large a proportion of the cases recovered. In thirty-two cases, only four proved fatal. These are all well attested cases; the name of the author is given, and there can be no doubt as to the correctness of the diagnosis in each instance. The operation of excision is more dangerous than that of ligature. Out of fourteen cases in which this operation was performed, four died. In a case of long-standing, I think I should prefer the ligature to manipulation, that is to say, if the patient was past the child-bearing period.

Dr. Quackenbush.—In rising to reply to some of the remarks of Professor Lee, perhaps I should offer an apology to the Society for trespassing so often on its time and attention in the discussion of this, to me, highly interesting subject. And yet, when I remember that I occupied the floor in replying to certain interrogatories proposed to me, I am the more ready to believe that the Society will pardon me if I again express my views in elucidation of some of the points to which the Professor has alluded. The subject of Inversion of the Uterus has lately attracted considerable attention in this country and in England, and its treatment seems now to be generally well understood. The cause of the difficulty, however, is not so definitely settled, and the *manner* of its occurrences is enveloped in much doubt and obscurity. It has generally, I might say universally, been supposed that the uterus become inverted by a depression of its fundus, that at this point there is an indentation which becomes larger and larger, and finally the whole organ turns inwards upon itself and becomes entirely inverted. This view is adopted by Professor Lee. I suggested, in a report read before this Society one year ago, another mode, namely, that the inversion of the organ commenced at the neck instead of the fundus—this the Professor characterizes as impossible; nay, as absurd! Upon this point, Mr. President, I take issue with my learned friend. Every gentleman present knows that if you place the hand on the abdomen of your patient shortly after the birth of the fœtus and placenta, the fundus feels hard and firm; while, if an examination be made *per vaginam*, the neck will be found to be flaccid and relaxed, and you can hardly tell where the vagina terminates and where the womb commences; and let me inquire, is not this the very condition we would expect to find if the inversion commenced in the manner indicated? But that I may be better understood, and that I may the better maintain my position, allow me to explain more fully the manner in which I think it occurs. It is well known that there are two layers of fibres in the uterus, one the circular or horizontal, the other the longitudinal layer; the former encircling as a band the os and cervix uteri, while the latter extends from this band and passes over the fundus of the uterus. When labor commences and proceeds, both these layers contract, but after a time the circular fibres yield to the more powerful action of the longitudinal, the os uteri opens, and the vagina and uterus become one continued and regular canal. The organic contractility continues, and the organ is freed from the uterus which it contained. Another contractility now comes into play. This is the contractility of the tissue, a property by which the womb, after having been emptied, returns gradually to its former state, and thereby has its cavity nearly obliterated. Now, at this stage there may be irregularity of contraction. The circular fibres, constituting a sort of sphincter muscle of the womb, are relaxed and form no firm attachment for the longitudinal fibres. The longitudinal fibres, which may represent so many columns resting on this circular band as a foundation, contract, and, having no support, they begin to yield from the bottom, evolution takes place, the neck doubles in upon itself and passes through the os, the body follows, and, finally, the fundus, dragged down upon the body, preserves the same course, and we now have a complete inversion; the fundus being the last portion inverted, instead of the first, as has been generally, or I may say, universally admitted.

Before leaving this point, I would state that I am not alone in my opinion in regard to this manner of evolution, for I find that Dr. John Delamater, who has had a very extensive practice, entertains the same views, and has made them public in a letter, written in April last. Allow me to read one paragraph. "The thought that inversion may, though rarely, perhaps, commence at the neck of the uterus, rather than at the fundus, does not appear to me to have occurred to any one but myself, and yet it is so clear to me that it must sometimes be so, that I have been compelled to argue the point" at much length. Mr. President, this manner of inversion seems not only plausible but extremely probable. True, all authors who have treated the subject teach differently, but what is the reason? They have seldom, if ever, seen the accident; their attention has not been particularly drawn to it; and hence they have adopted this universal notion, which, in my opinion, is as erroneous as it is universal. If then this be the manner in which inversio uteri occurs, what is the cause of it? I consider it an irregularity of action, and by this term I do not mean an undue action of some of the fibres of the body and fundus, whereby a portion is drawn down or depressed, but a *want of correspondence* between the muscular action of the neck and the body of the uterus, in which there is a complete atony of the muscular fibres of the neck, which is consequently soft and yielding, and a partial action of the fibres of the body and fundus, sufficiently strong to draw it down through the patulous mouth of the womb, but not active enough to detach the placenta, which we usually find adherent. Here, then, we have an atonic and patulous condition of the os and cervix uteri, affording no impediment to the protrusion of the body and fundus, which is drawn down by the slight muscular contractions, by the traction on the cord, by the weight itself of the fundus with the placenta attached, or perhaps pushed down by the superincumbent mass of intestines, aided by the contraction of the abdominal muscles. I am led to this conclusion by the fact that in numerous cases of this character the placenta remains attached to the uterus, not only after it is inverted, but even when it has protruded through the vulva, which would not be the case if the action had been excessive, for the contractions, violent enough to produce this inverted condition of the organ, would certainly be sufficiently powerful to detach the placenta.

There are other points, Mr. President, which I would be pleased to discuss, as the subject is full of interest, but I do not feel at liberty to take up any more of the time and the attention of the Society at this session.

On motion, the meeting adjourned until 10 A. M. on Wednesday morning.

